# Lipoprotein lipase hydrolysis products induce pro-inflammatory cytokine expression in triple-negative breast cancer cells

**DOI:** 10.1186/s13104-021-05728-z

**Published:** 2021-08-17

**Authors:** Alexandria J. Tobin, Nicholas P. Noel, Sherri L. Christian, Robert J. Brown

**Affiliations:** Department of Biochemistry, Memorial University of Newfoundland, 232 Elizabeth Ave., St. John’s, NL A1B 3X9 Canada

**Keywords:** Lipoprotein lipase, Lipoproteins, Breast cancer, Cytokines, Metabolic activity, Antibody arrays

## Abstract

**Objectives:**

Breast cancer cell growth and proliferation requires lipids for energy production, cell membrane synthesis, or as signaling molecules. Lipids can be delivered to cells by lipoprotein lipase (LPL), an extracellular lipase that hydrolyzes triacylglycerols and phospholipids from lipoproteins, that is expressed by adipose tissue and some breast cancer cell lines. Studies have shown that lipoprotein hydrolysis products induce pro-inflammatory cytokine secretion by endothelial cells. Thus, our objective was to determine if hydrolysis products generated by LPL from total lipoproteins can also promote pro-inflammatory cytokine secretion from breast cancer cells.

**Results:**

Using cytokine arrays, we found that MDA-MB-231 cells increased secretion of seven cytokines in response to treatment with lipoprotein hydrolysis products. In contrast, MCF-7 cells showed decreased secretion of two cytokines. Expanding the analysis to additional cell lines by ELISA, we found increased secretion of TNF-α and IL-6 by MDA-MB-468 cells, and increased secretion of IL-4 by MDA-MB-468 and SKBR3 cells. The changes to cytokine secretion profiles of the breast cancer cell types examined, including the non-cancerous MCF-10a breast cells, were independent of increased cell metabolic activity. These results provide information on how lipoprotein hydrolysis products within the tumor microenvironment might affect breast cancer cell viability and progression.

**Supplementary Information:**

The online version contains supplementary material available at 10.1186/s13104-021-05728-z.

## Introduction

Breast cancer is the most diagnosed cancer in women and a leading cause of death from cancer in women [1]. Clinically, breast cancer is diagnosed based on expression levels of the estrogen receptor (ER), progesterone receptor (PR), and human epidermal growth receptor 2 (HER2), yielding three main types of breast cancer: ER+/PR+, HER2+, and triple-negative—each with its own treatment strategies and prognosis [2]. Contributing to the growth and proliferation of breast cancer cells is the presence of lipoprotein lipase (LPL) in their microenvironment, resulting in an increased delivery of free fatty acids (FFA) for energy production via β-oxidation [3].

LPL is an extracellular cell surface-associated *sn*-1 lipase that hydrolyzes ester bonds within triacylglycerols and phospholipids that are carried by lipoproteins, to yield FFA, mono- and diacylglycerols from triacylglycerols, and lysophospholipids from phospholipids [4]. In addition, LPL has a non-catalytic function where lipoproteins are captured to bridge them to cell surface receptors, thus promoting lipid uptake by cells [5]. LPL is highly expressed in the adipose tissue, skeletal muscle, and cardiac muscle, and in other tissues, including mammary tissue [6]. *LPL* gene expression has been detected in select human breast cancer cell lines, and LPL protein and activity were identified in the Du4475 cell line and primary breast tumor tissues [7].

Of note, LPL hydrolysis products generated from very low-density lipoproteins were shown to increase tumor necrosis factor (TNF)-α secretion from endothelial cells [8], and to increase intercellular adhesion molecule-1 (ICAM-1) expression on endothelial cells [8]. In breast cancer, ICAM-1 is upregulated by TNF-α and it is thought that ICAM-1 is involved in tumor cell invasion and metastasis by promoting intravasation [9]. However, the role of lipoprotein hydrolysis products generated by LPL on cytokine secretion in breast cancer remains to be investigated. Thus, for the current study, our objective was to examine the cytokine expression profiles of breast cancer cells with differing ER/PR and HER2 receptor status, in response to total lipoprotein hydrolysis products generated by LPL. We also investigated whether these changes were associated with alterations to metabolic activity.

## Main text

### Methodology

Lipoprotein hydrolysis products were generated by LPL (or no LPL—mock) using total lipoproteins from normolipidemic human plasma. Hydrolysis products were diluted to 0.68 mM and incubated for 24 h with human breast cancer cells (MDA-MB-231, MDA-MB-468, SKBR3, MCF-7, and T47D) or MCF-10a non-tumorigenic human breast cells. Conditioned media from cells were examined for cytokines using cytokine arrays and/or ELISAs. Cell metabolic activity was examined using an MTT assay. See Additional file 1: detailed methodology.

### Results

#### Antibody array analyses of cytokines from MDA-MB-231 and MCF-7 cell lines in response to LPL hydrolysis products

**Fig. 1:**
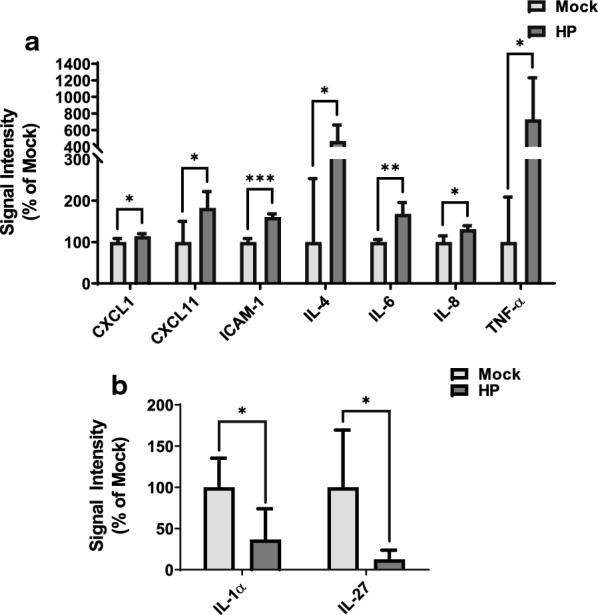
Cytokine array analysis of MDA-MB-231 and MCF-7 cell supernatants following treatment of cells with total lipoprotein lipid hydrolysis products generated by LPL. Cells were treated with either total lipoprotein lipid hydrolysis products generated by LPL (HP) or mock heparinized media (Mock) for 24 h. Conditioned media from MDA-MB-231 cells (**A**) and MCF-7 cells (**B**) were examined for cytokines using an antibody array. Signal intensities were obtained by scanning densitometry, normalized to an internal control within the array, and presented as a percent of Mock. Data are presented as the mean of triplicate biological experiments, ± SD. *p < 0.05; **p < 0.01; ***p < 0.001

Our objective was to examine cytokine secretion profiles from different breast cancer cell lineages in response to total lipoprotein hydrolysis products generated by LPL. To test this, using antibody arrays, we examined the secretion levels of 36 different cytokines (Additional file 2: Table S1) in the supernatants of triple-negative breast cancer (TNBC) MDA-MB-231 cells, and ER+/PR+/HER2− MCF-7 cells, treated for 24 h with either hydrolysis products generated by LPL, or mock control media (without LPL). We observed differing cytokine secretion profiles between the two cell types. Across three independent experiments, seven cytokines were significantly increased in media from hydrolysis product-treated MDA-MB-231 cells: CXC motif chemokine ligand (CXCL) 1, CXCL11, ICAM-1, interleukin (IL)-4, IL-6, IL-8, and TNF-α (Fig. 1A). No detection or no changes were observed with other cytokines. These cytokines did not change in MCF-7 cells in response to hydrolysis products; however, two cytokines exhibited a significant decrease: IL-1α and IL-27 (Fig. 1B).

#### ELISA analyses of cytokines from breast cancer cell lines in response to LPL hydrolysis products

**Fig. 2:**
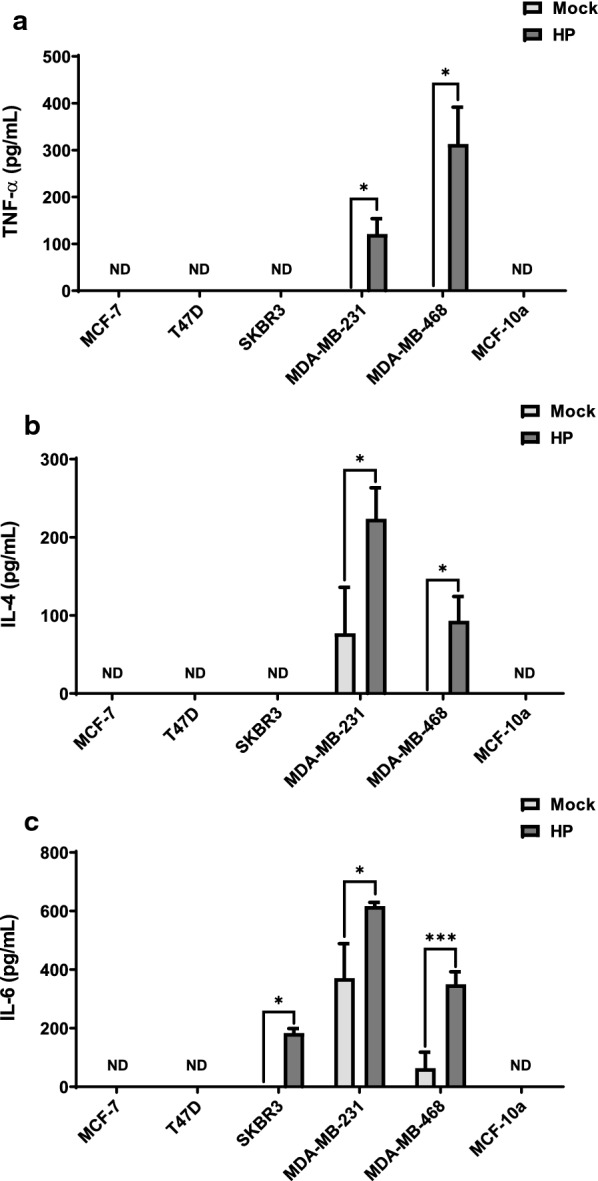
TNF-α, IL-4, and IL-6 expression by ELISA of breast cancer and MCF-10a cell supernatants following treatment with total lipoprotein lipid hydrolysis products generated by LPL. Breast cancer cells and MCF-10a cells were treated with either total lipoprotein lipid hydrolysis products generated by LPL (HP) or mock heparinized media (Mock) for 24 h. Conditioned media were examined for (**A**) TNF-α, (**B**) IL-4, and (**C**) IL-6 by ELISA. Data are presented as the mean of triplicate biological experiments, ± SD. *ND* not detected; *p < 0.05; **p < 0.01; ***p < 0.001

Because our array data suggested that breast cancer cell receptor status results in differing cytokine secretion profiles in response to hydrolysis products, we expanded our analysis to additional cell lines. Using ELISA, we specifically analyzed the secretion of TNF-α, IL-4, and IL-6 by MCF-7, T47D (ER+/PR+/HER2−), SKBR3 (ER−/PR−/HER2+), MDA-MB-231, MDA-MB-468 (TNBC), and MCF-10a (control non-tumorigenic mammary epithelial) cells, treated for 24 h with hydrolysis products (or mock control). TNF-α was only detected in conditioned media from MDA-MB-231 and MDA-MB-468 cells treated with hydrolysis products (Fig. 2A). These data are consistent with the expression pattern of the TNF-α data obtained for MDA-MB-231 and MCF-7 cells by the cytokine arrays. Compared to control, IL-4 was significantly increased in conditioned media from MDA-MB-231 cells treated with hydrolysis products (Fig. 2B). IL-4 was not detected in conditioned media from control-treated MDA-MB-468 cells, but it was present in the media of MDA-MB-468 cells treated with hydrolysis products (Fig. 2B). No other cell lines tested had detectable levels of IL-4 in their media, regardless of treatment (Fig. 2B). For MDA-MB-231 and MDA-MB-468 cells, IL-6 was significantly increased in the conditioned media from cells treated with hydrolysis products compared to control (Fig. 2C). The levels of IL-6 were also significantly increased in the conditioned media from SKBR3 cells treated with hydrolysis products compared to control (Fig. 2C). However, media from MCF-7, T47D, and MCF-10a cell lines did not have any detectable IL-6, regardless of treatment (Fig. 2C).

#### Metabolic activities of breast cancer cell lines in response to LPL hydrolysis products

**Fig. 3:**
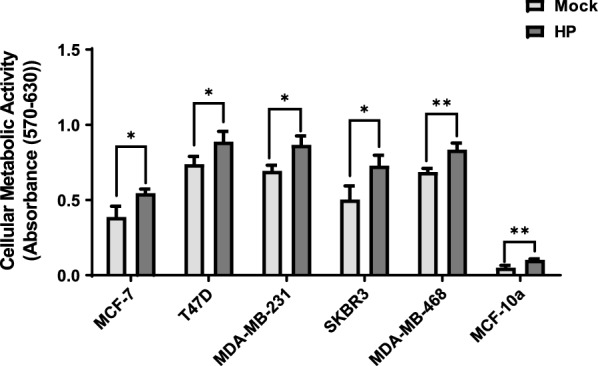
MTT assay of breast cancer and MCF-10a cells following treatment with total lipoprotein lipid hydrolysis products generated by LPL. MCF-7, T47D, SKBR3, MDA-MB-231, MDA-MB-468 breast cancer cells and MCF-10a cells were treated with either total lipoprotein lipid hydrolysis products generated by LPL (HP) or mock heparinized media (Mock) for 24 h. At 4 h after the addition of MTT, the absorbance of the samples was read at 570 nm and 630 nm. Cellular metabolic activity was calculated by subtracting the 630 nm data from the 570 nm data. Data are presented as the mean of triplicate biological experiments with triplicate wells assessed per experiment, ± SD. *p < 0.05; **p < 0.01

We sought to determine if differing cytokine profiles were related to metabolic activity. To determine the effects of hydrolysis products on metabolic activities of breast cancer cells of different subtypes, MCF-7, T47D, SKBR3, MDA-MB-231, MDA-MB-468 and MCF-10a cells were cultured and treated for 24 h with hydrolysis products, or with mock control media. The metabolic activity of all cell lines treated with LPL-generated hydrolysis products was significantly increased by approximately 20% compared to mock control (Fig. 3). Overall, these data suggest that cytokine expression profiles in response to hydrolysis products from total lipoproteins are independent of alterations to metabolic activity.

### Discussion

Breast cancer cells can secrete cytokines to induce changes in the surrounding cells, and similarly, cells of the microenvironment can secrete cytokines to support or reduce cancer survival [10]. Surprisingly, the cytokine profiles of conditioned media from MDA-MB-231 and MCF-7 cells in the presence of lipoprotein hydrolysis products were quite different (Additional file 3: Fig. S1). These cell lines were chosen as they represent the most and least aggressive breast cancer subtypes, respectively. This observation, combined with the observation that media from the MDA-MB-231 cells had increased levels of several pro-tumorigenic cytokines in the presence of lipoprotein hydrolysis products, led us to examine additional cell lines for levels of secreted TNF-α, IL-4, and IL-6.

The secretion profile of the TNBC cell lines we examined in the presence of lipoprotein lipid hydrolysis products is pro-tumorigenic, with significant levels of secreted TNF-α, IL-4, and IL-6 compared to controls. Breast cancer cells can produce and utilize TNF-α to activate nuclear factor (NF)-κB signaling. NF-κB activation can induce tumor cell proliferation, angiogenesis, immune evasion, and metastasis [11, 12]. One result of NF-κB activation is increased expression of IL-8, which was also found to be significantly increased using the cytokine array. IL-8 promotes angiogenesis, tumor cell migration, and immune cell infiltration in breast cancer [13]. Similarly, NF-κB activation by TNF-α increases ICAM-1 expression in breast cancer, which is upregulated to promote metastasis and is associated with more aggressive subtypes [9]. Unsurprisingly, the cytokine array data revealed a significant increase in secreted ICAM-1 by MDA-MB-231 cells, which suggests that hydrolysis products may promote development of a more aggressive tumor phenotype. CXCL1, which promotes immune cell invasion and angiogenesis, was also found to be upregulated. Interestingly, CXCL1 is also induced by TNF-α. Thus, it is possible that TNF-α induces the secretion of more pro-inflammatory cytokines via the activation of NF-κB, although this remains to be determined.

In contrast, IL-4 has been described as an anti-inflammatory cytokine that induces apoptosis in breast cancer cells [14]. However, IL-4 secretion activates M2-like tumor-associated macrophages, which are strongly pro-tumorigenic [15]. The pleiotropic cytokine IL-6 was also increased in response to hydrolysis products. IL-6 is involved in multiple aspects of tumor progression, including invasion, metastasis, angiogenesis, and resistance to therapy. The broad impact of IL-6 is due to its ability to activate many different signaling pathways that have pro-tumorigenic functions, such as the NF-κB [8] and JAK/STAT3 [16] pathways. Finally, increased secretion of CXCL11 in MDA-MB-231 cells was unexpected. CXCL11 attracts mononuclear immune cells to the tumor microenvironment. Once in the tumor microenvironment, the immune cells may have a pro- or anti-tumorigenic effect depending on context. CXCL11 is primarily induced by interferon (IFN)-γ [17], yet IFN-γ secretion was not detected in this study possibly because it was below detectable levels. Taken together, these data suggest that hydrolysis products liberated by LPL from total lipoproteins promote tumorigenesis by upregulating pro-tumorigenic cytokine secretion in TNBC cells.

The cytokine array data for conditioned media from MCF-7 cells are more difficult to interpret. MCF-7 cells only showed a significant decrease in IL-1α and IL-27. IL-1α is a pro-inflammatory cytokine that can promote cancer progression by activating NF-κB and JAK/STAT3 signaling. Because of the pro-tumorigenic effects of IL-1α [18], it would be expected that IL-1α would be upregulated in breast cancer cells following hydrolysis products treatment. However, IL-1α has been shown to inhibit MCF-7 cell proliferation by causing cell cycle arrest [19]. This is an interesting and counterintuitive observation because it indicates that IL-1α downregulation by lipoprotein hydrolysis products may function to sustain tumorigenesis in MCF-7 cells. Similarly, IL-27 is known to have both pro- and anti-tumorigenic effects. IL-27 can induce T cell activation in the tumor microenvironment, resulting in different effects depending on the degree of progression. IL-27 can also activate STAT1, which has potent anti-proliferative effects, induces apoptosis, and enhances immune cell elimination of cancer cells [20]. Like IL-1α, IL-27 downregulation by lipoprotein hydrolysis products may be pro-tumorigenic in our model. Because of their multifunctional roles, IL-1α and IL-27 likely have both pro- and anti-inflammatory effects in vivo. However, these data suggest that LPL hydrolysis products may promote tumorigenesis in ER+/PR+ luminal A breast cancer.

The TNBC cell lines exhibited a clear pro-tumorigenic phenotype in response to hydrolysis products from total lipoproteins generated by LPL, and the changes to IL-1α and IL-27 in MCF-7 cells may lean toward a pro-tumorigenic phenotype in ER+/PR+ cells. T47D cells, like MCF-7 cells, are ER+/PR+/HER2−. While we did not examine the T47D cell line in the cytokine array, like the MCF-7 cells, we did not detect secreted TNF-α, IL-4, and IL-6, regardless of treatment; however, we anticipate that T47D cells may also show changes to IL-1α and IL-27 expression—a hypothesis which will be addressed in future experiments. The ER−/PR−/HER2 + SKBR3 cell line did release excess IL-6 in the presence of lipoprotein hydrolysis products, but not TNF-α or IL-4.

Collectively, our data suggest that components within total lipoprotein lipid hydrolysis products generated by LPL change the cytokine secretion profile of breast cancer cells in a subtype-specific manner. At least for the MDA-MB-468 cell line, it appears that one or more components within the FFA component may be responsible for directly affecting TNF-α expression, as our data show a 12.4-fold increase in *TNFA* mRNA expression compared to control treatments (Additional file 4: Fig. S2). This observation is similar to what we observed with THP-1 macrophages [21].

### Conclusions

Our study shows that products of lipid hydrolysis from total lipoproteins by LPL may promote breast cancer growth and progression by inducing a pro-tumorigenic cytokine secretion profile from tumor cells, independent of cell metabolic activity. Of interest, the lipoprotein hydrolysis products induce different pro-tumorigenic cytokine expression profiles in different breast cancer subtypes. This suggests that lipoprotein hydrolysis products have distinct effects between breast cancer cell subtypes, that could result in promoting breast cancer progression.

### Limitations

Our data reflect changes to cytokine expression using hydrolysis products from lipoproteins obtained from normolipidemic subjects. It is possible that lipoproteins from subjects who are obese, hyperlipidemic, or on long-term dietary interventions may yield differing outcomes. In addition, analyses of patient-derived breast cancer cells are needed to determine how cytokine secretion is affected in primary cells. These limitations will need to be addressed in future experiments.

## Supplementary Information

**Additional file 1**: Detailed methodology.

**Additional file 2: Table S1.** Cytokines examined using the Proteome Profiler™ Human Cytokine Array.

**Additional file 3: Fig. S1.** Schematic diagram of antibody array results. The cytokine profiles of MDA-MB-231 and MCF-7 cells in response to total lipoprotein lipid hydrolysis products generated by LPL point toward a pro-tumorigenic phenotype. Using antibody arrays, we showed that the concentrations of seven cytokines increased in the media from TNBC MDA-MB-231 cells in response to hydrolysis products; each of these cytokines have pro-tumorigenic properties. In contrast, two cytokines exhibited decreased concentrations from the media of ER + /PR + /HER2- MCF-7 cells treated with hydrolysis products. However, the downregulation of these two cytokines may also have pro-tumorigenic effects.

**Additional file 4: Fig. S2.** Expression of *TNFA* in MD-MBA-468 cells in response to the FFA component of total lipoprotein hydrolysis products generated by LPL. The FFA component that is generated from the hydrolysis of lipoprotein lipids by LPL was reconstituted as previously described—see Additional file 1: Detailed methodology. MDA-MB-468 cells were treated with either the FFA component or vehicle control for 18 h, as previously described—see Additional file 1: Detailed methodology. Following treatment, RNA was extracted from cells and examined for the expression of *TNFA* and normalized against the expression data for *ACTB*. Primer information and qPCR conditions were previously reported—see Additional file 1: Detailed methodology. Data are the average ± SD of two biological experiments.

## Data Availability

All data generated and analyzed during this study are included in this article and its supplementary information files. All data may be requested from the corresponding authors upon reasonable request.

## Abbreviations

CXCL: CXC motif chemokine ligand
ER: Estrogen receptor
FFA: Free fatty acid
HER2: Human epidermal growth factor 2
ICAM-1: Intercellular adhesion molecule-1
IFN: Interferon
IL: Interleukin
LPL: Lipoprotein lipase
NF: Nuclear factor
PR: Progesterone receptor
TNBC: Triple-negative breast cancer
TNF: Tumor necrosis factor
