# Supplementary material for: Lipoprotein lipase hydrolysis products induce pro-inflammatory cytokine expression in triple-negative breast cancer cells
Source: BMC Res Notes. 2021 Aug 17;14:315. doi: 10.1186/s13104-021-05728-z (PMC8369739; doi:10.1186/s13104-021-05728-z)
Supplement: Supplementary file 1 — Additional file 1: Detailed methodology. [file 13104_2021_5728_MOESM1_ESM.pdf]

Detailed methodology

Cell lines

Human embryonic kidney (HEK-293) cells and the human breast cancer cell lines MDA-MB-231, MDA-MB-468, and SKBR3 were obtained from the American Type Culture Collection (ATCC), and maintained at 37°C with 5% $CO_{2(g)}$ in Dulbecco's Modified Eagle Medium (DMEM) (Fisher Scientific), supplemented with 10% v/v fetal bovine serum (FBS) (Sigma-Aldrich) and 1% v/v antibiotic/antimycotic (A/A) (Sigma-Aldrich). MCF-7 and T47D human breast cancer cell lines (ATCC) were maintained at 37°C with 5% $CO_{2(g)}$ in Roswell Park Memorial Institute (RPMI)-1640 medium (ThermoFisher Scientific), supplemented with 10% v/v FBS and 1% v/v A/A. MCF-10a non-tumorigenic human breast cells (ATCC) were maintained at 37°C with 5% $CO_{2(g)}$ in DMEM/F-12 medium (ThermoFisher Scientific), supplemented with 5% v/v horse serum (ThermoFisher Scientific), 20 ng/mL epidermal growth factor (EGF) (ThermoFisher Scientific), 10 µg/mL insulin (ThermoFisher Scientific), 0.5 µg hydrocortisone (ThermoFisher Scientific), and 1% v/v A/A. To authenticate each cell line examined, STR profiling of each cell line was carried out by The Centre for Applied Genomics (Hospital for Sick Children, Toronto). Prior to use, all cell lines were confirmed to be mycoplasma free using the MycoAlert™ PLUS Mycoplasma Detection Kit (Lonza).

LPL expression

LPL was expressed in HEK-293 cells and released from cell surfaces using heparin, as previously described [1,2]. Briefly, at 70-80% confluency in 10 cm dishes, cells were transfected with either 5.85 µg of a pcDNA3 vector containing human LPL cDNA [Gen-Bank: NM_000237], or no plasmid (mock), using Lipofectamine™ (ThermoFisher Scientific) per the manufacturer's protocol. Our laboratory has previously reported no difference in lipase activity between control cells transfected with an empty pcDNA3 vector and cells transfected with no vector [3]. Following 24 h of incubation, the media were removed and replaced with 5 ml of DMEM medium containing 1% v/v A/A and 10 U/ml heparin (Organon). After 23.5 h of incubation, 1 ml of DMEM containing 100 U/ml heparin and 1% v/v A/A was added. After 30 min, media were collected and centrifuged at 1,000 ×*g* for 10 min to remove cell debris. Supernatants were aliquoted into microfuge tubes and stored at -80˚C until needed. The presence of LPL was validated by immunoblot analysis with a polyclonal anti-human LPL antibody (#sc-32885, Santa Cruz Biotechnology) as previously described [1], except at a 1:2,000 dilution, and the examination of catalytic activity toward 1,2-*O*-dilauryl-*rac*-3-glutaric-resorufin ester (Sigma-Aldrich) was used to measure the enzymatic activity of LPL as previously described [4]. A single band of 58 kDa was observed in the heparinized media from cells expressing LPL, but not in the heparinized media from mock transfected cells (data not shown). The activity of the heparinized media from cells expressing LPL was 2.97 ± 0.63 µmol/ml/min, versus 0.20 ± 0.02 µmol/ml/min for heparinized media from mock transfected cells.

Lipoprotein hydrolysis products
Plasma from three anonymous normolipidemic donors fasted overnight were collected and pooled to isolate total lipoproteins ($\rho<1.21$ g/mL). Lipoproteins were isolated using a KBr density gradient coupled with ultracentrifugation [5]. The phospholipid content of the total lipoproteins was quantified using the Wako Phospholipid C assay kit (Wako Diagnostics), according to manufacturer's instructions. To generate lipoprotein hydrolysis products, lipoproteins were diluted to a phospholipid concentration of 3.5 mM, then were incubated in a 1:1 ratio of total lipoproteins with either heparinized medium containing LPL, or heparinized medium containing no LPL (mock), at 37°C for 4 h. After incubation, the FFA generated were quantified using the Wako NEFA-HR(2) assay kit (Wako Diagnostics), according to manufacturer's instructions. The FFA generated using the heparinized media from cells expressing LPL was 1.30 ± 0.01 nmol/µl/4h, versus 0.07 ± 0.01 nmol/µl/4h for heparinized media from mock transfected cells.

Treatment of cells with lipoprotein hydrolysis products
For the assessment of metabolic activity in response to lipoprotein hydrolysis products using the 3-(4, 5-dimethylthiazolyl-2)-2,5-diphenyltetrazolium bromide (MTT) assay, breast cancer and MCF-10a cell lines were plated at 2.3×10^4 cells/well within 96-well plates. For the assessment of media cytokines, breast cancer and MCF-10a cell lines were plated at 9.7×10^5 cells/well within 6-well plates. At 24 h after plating, cells were pre-treated for 1 h with a fatty acid-free medium solution consisting of 0.2% w/v fatty acid-free bovine serum albumin (FAF-BSA) (Sigma-Aldrich), 25 µg/ ml tetrahydrolipstatin (THL) (Sigma-Aldrich), and 1% v/v A/A within DMEM (for MDA-MB-231, MDA-MB-468, and SKBR3 cells), RPMI-1640 (for MCF-7 and T47D cells), or DMEM/F-12 (for MCF-10a cells). For 96-well plates, 150 µl/well of media were used; for 6-well plates, 1 ml of media were used. After 1 h, media were replaced with the FAF-BSA-containing media with either 0.68 mM lipoprotein hydrolysis products (by FFA content), or with heparinized media from mock-transfected cells, comparably diluted (by volume). After 24 h of incubation in the absence or presence of lipoprotein hydrolysis products, cells were examined for metabolic activity or for cytokine expression.

MTT assay
MTT (Fisher Scientific) was dissolved in phosphate-buffered saline (pH 7.4) to make a 5 mg/mL solution. Following hydrolysis product (or mock) treatments of cells within 96-well plates, 50 µl of conditioned media were removed (for future analyses), and 10 µl of MTT added to each well, followed by a 4 h incubation at 37°C. After 4 h, formazan crystals that formed were dissolved by adding 100 µl of 0.1 N HCl in isopropanol (ThermoFisher Scientific) to each well with thorough mixing. The absorbance of each well was read at 570 nm and 630 nm; the 630 nm values were subtracted from the 570 nm data to account for background noise. The final values are proportional to the metabolic activity of the cell.

Cytokine arrays and cytokine enzyme-linked immunoassays

The presence and relative levels of various cytokines in the conditioned media of MDA-MB-231 and MCF-7 cells, treated with lipoprotein hydrolysis products or mock control media, were assessed using the Proteome Profiler™ Human Cytokine Array kit (R&D Systems), according to the manufacturer's instructions. One ml of medium was mixed with 0.5 ml Array Buffer 4 (from the kit), and 15 µl of a human cytokine array detection antibody cocktail (from the kit); each supernatant mixture was applied to its own multiplexed array. Chemiluminescence from the arrays was captured digitally using an ImageQuant LAS detection system. ImageJ software [6] was used to obtain pixel density values of each spot of the array. Duplicate spots for each cytokine were averaged, and pixel density data were normalized to the reference spots on the array. All array data were presented as a percent of mock control treatment data.

The concentrations of tumor necrosis factor (TNF)-α, interleukin (IL)-4, and IL-6 of breast cancer and MCF-10a cells incubated in the absence or presence of lipoprotein hydrolysis products were examined by enzyme-linked immunoassay (ELISA), using the Human TNF-α, Human IL-4, or Human IL-6 DuoSet® ELISA development systems, respectively (R&D Systems), per manufacturer's instructions but with the following minor changes. Recombinant TNF-α (at 15.6, 31.3, 62.5, 125, 250, 500, or 1,000 pg/ml), IL-4 (at 31.3, 62.5, 125, 250, 500, 1000, 2,000 pg/ml), and IL-6 (at 9.38, 18.8, 37.5, 75, 150, 300, 600 pg/ml) were used to obtain standard curves. Conditioned media were diluted 1:10, 1:100, and 1:1,000. Following blocking of each well, 200 µl of diluted conditioned medium was used for each well. Ultra TMB-ELISA substrate solution (100 µl/well, ThermoFisher Scientific) was used for detection. Color development was stopped by the addition of 50 µl of 2 M H_2SO_4. The absorbance was immediately read at 450 nm and 540 nm; the data obtained at 540 nm were subtracted from the 450 nm data, to correct for background noise.

Real-time qPCR

The FFA component of total lipoprotein hydrolysis products generated by LPL was previously reported [1], and reconstituted using purified FFA (Nu-Chek Prep) in DMSO as previously described [3]. The FFA mixture (containing 18.6 nmol of myristate, 275.0 nmol of palmitate, 23.7 nmol of palmitoleate, 45.4 nmol of stearate, 241.8 nmol of oleate, 70.0 nmol of linoleate, 0.9 nmol of arachidonate, and 0.4 nmol of docosahexaenoate in 10 µl DMSO), or DMSO vehicle control, were incubated with 9.7×10^5 MDA-MB-468 cells/well within 6-well plates, as previously described [3]. To make 1 ml of tissue culture media for the incubations, the FFA/DMSO mixture or 10 µl DMSO (as vehicle control) was added at a rate of 1 µl/min to 990 µl of media (appropriate to the cell line, as noted above for lipoprotein hydrolysis products), containing 0.2% w/v FAF-BSA, and 1% v/v A/A while continuously vortexing. After 18 h, RNA was extracted using TRIzol (ThermoFisher Scientific), following manufacturer's instructions. cDNA was synthesized from 500 ng of RNA using M-MLV reverse transcriptase (ThermoFisher Scientific) with random hexamers (#48190-011, ThermoFisher Scientific), according to manufacturer's instructions. Quantitative real-time PCR was performed on a CFX96 Touch™

Real-Time PCR System (Bio-Rad), using iQ SYBR Green Supermix (Bio-Rad), according to manufacturer's instructions. Primers for *TNFA* were purchased as a forward/reverse mix (#qHSACED0037461, Bio-Rad), and these were determined to have an amplification efficiency of 0.98, calculated as previously described [7]. Primers for *ACTB* were 5'-ACC TTC TAC AAT GAG CTG CG-3' (forward), 5'-CCT GGA TAG CAA CGT ACA TGG-3' (reverse); these were determined to have an amplification efficiency of 0.73. Real-time PCR cycle conditions were 1 cycle of 95°C for 3 min, and 40 cycles of 95°C for 15 s, 59.5°C for 15 s, and 72°C for 20 s. All data were normalized to *ACTB* expression, and the relative expression ratio was calculated as previously described [7]. Data are presented as mean percentage relative to control treatments.

Statistical analyses

Statistical analyses were performed within GraphPad Prism 9.0 using either an unpaired Student's t-test for two groups or one-way ANOVA followed by Tukey's post-hoc test for more than two groups. All experiments were carried out as biological triplicates, with at least duplicate replicates within each experiment. All data are shown as mean ± SD, with significance assigned to differences with a $p<0.05$.

References

1. Essaji Y, Yang Y, Albert CJ, Ford DA, Brown RJ. Hydrolysis products generated by lipoprotein lipase and endothelial lipase differentially impact THP-1 macrophage cell signalling pathways. Lipids. 2013;48:769-78.
2. Yang Y, Thyagarajan N, Coady BM, Brown RJ. Cholesterol efflux from THP-1 macrophages is impaired by the fatty acid component from lipoprotein hydrolysis by lipoprotein lipase. Biochem Biophys Res Commun. 2014;451:632-6.
3. Thyagarajan N, Marshall JD, Pickett AT, Schumacher C, Yang Y, Christian SL, Brown RJ. Transcriptomic analysis of THP-1 macrophages exposed to lipoprotein hydrolysis products generated by lipoprotein lipase. Lipids. 2017;52:189-205.
4. Lehner R, Verger R. Purification and characterization of a porcine liver microsomal triacylglycerol hydrolase. Biochemistry. 1997;36:1861-8.
5. Chung BH, Wilkinson T, Geer JC, Segrest JP. Preparative and quantitative isolation of plasma lipoproteins: rapid, single discontinuous density gradient ultracentrifugation in a vertical rotor. J Lipid Res. 1980;21:284-91.
6. Schneider CA, Rasband WS, Eliceiri KW. NIH Image to ImageJ: 25 years of image analysis. Nat Methods. 2012;9:671-5.
7. Pfaffl MW. A new mathematical model for relative quantification in real-time RT-PCR. Nucleic Acids Res 2001;29:e45.
