# Supplementary material for: Lipoprotein lipase hydrolysis products induce pro-inflammatory cytokine expression in triple-negative breast cancer cells
Source: BMC Res Notes. 2021 Aug 17;14:315. doi: 10.1186/s13104-021-05728-z (PMC8369739; doi:10.1186/s13104-021-05728-z)
Supplement: Supplementary file 2 — Additional file 2: Table S1. Cytokines examined using the Proteome Profiler™ Human Cytokine Array. [file 13104_2021_5728_MOESM2_ESM.pdf]

Table S1: Cytokines examined using the Proteome Profiler^{TM} Human Cytokine Array

**Molecules**
Chemokine (C-C motif) ligand 1, 2, and 5
Chemokine (C-X-C motif) ligand 1, 10, 11, and 12
Cluster of differentiation 40 ligand
Complement component 5
Granulocyte-colony stimulating factor
Granulocyte-macrophage-colony stimulating factor
Intercellular adhesion molecule-1
Interferon-γ
Interleukin-1α, -1β, -2, -4, -5, -6, -8, -10, -12, -13, -16, -17A, -17E, -18, -21, -27, and -32α
Interleukin-1 receptor agonist
Macrophage inflammatory protein-1α
Macrophage migration inhibitory factor
Serpin E1
Triggering receptor expressed on myeloid cells-1
Tumor necrosis factor-α
