# Supplementary material for: Lipoprotein lipase hydrolysis products induce pro-inflammatory cytokine expression in triple-negative breast cancer cells
Source: BMC Res Notes. 2021 Aug 17;14:315. doi: 10.1186/s13104-021-05728-z (PMC8369739; doi:10.1186/s13104-021-05728-z)
Supplement: Supplementary file 3 — Additional file 3: Fig. S1. Schematic diagram of antibody array results. The cytokine profiles of MDA-MB-231 and MCF-7 cells in response to total lipoprotein lipid hydrolysis products generated by LPL point toward a pro-tumorigenic phenotype. Using antibody arrays, we showed that the concentrations of seven cytokines increased in the media from TNBC MDA-MB-231 cells in response to hydrolysis products; each of these cytokines have pro-tumorigenic properties. In contrast, two cytokines exhibited decreased concentrations from the media of ER + /PR + /HER2- MCF-7 cells treated with hydrolysis products. However, the downregulation of these two cytokines may also have pro-tumorigenic effects. [file 13104_2021_5728_MOESM3_ESM.pdf]

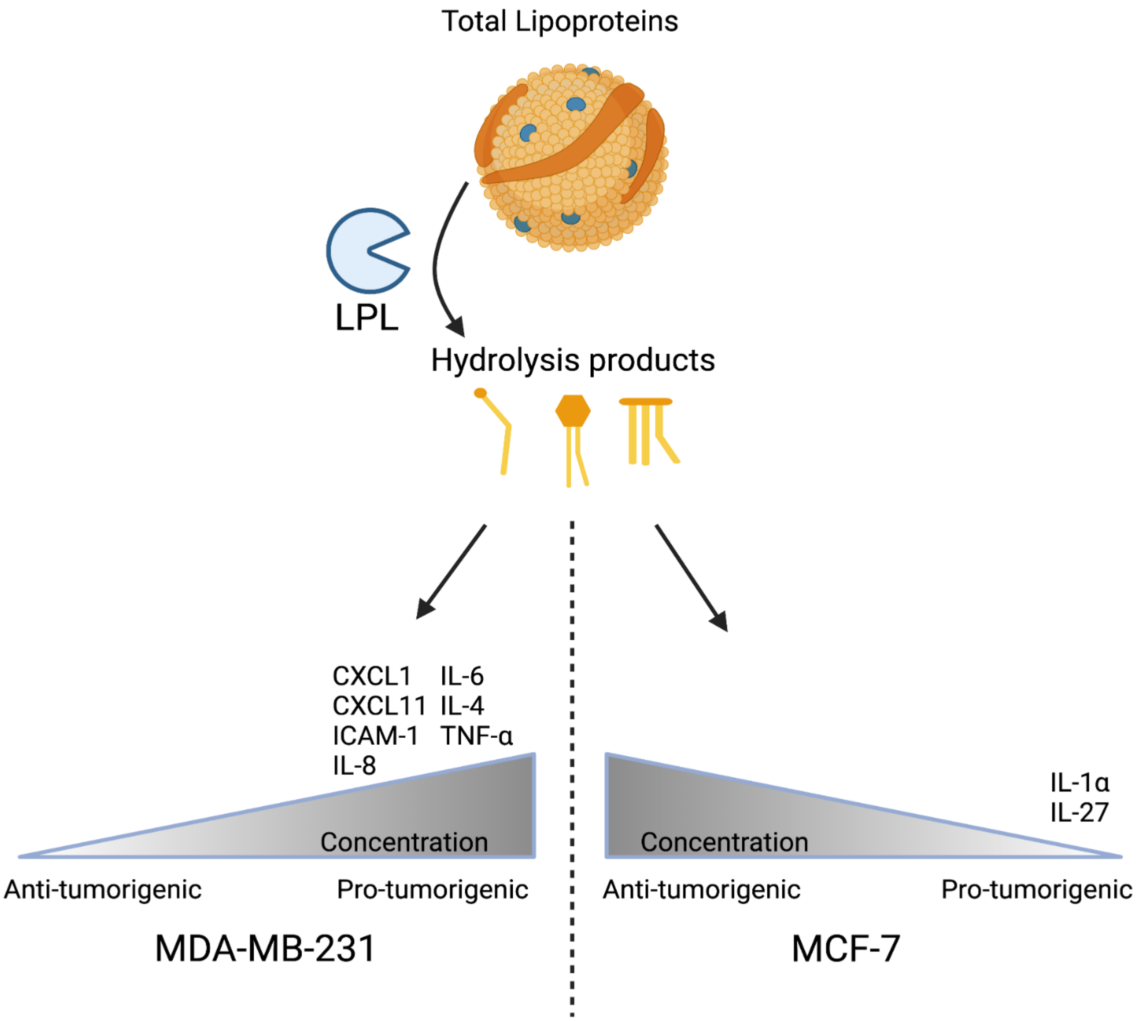

Figure S1. Schematic diagram of antibody array results. The cytokine profiles of MDA-MB-231 and MCF-7 cells in response to total lipoprotein lipid hydrolysis products generated by LPL point toward a pro-tumorigenic phenotype. Using antibody arrays, we showed that the concentrations of seven cytokines increased in the media from TNBC MDA-MB-231 cells in response to hydrolysis products; each of these cytokines have pro-tumorigenic properties. In contrast, two cytokines exhibited decreased concentrations from the media of ER+/PR+/HER2- MCF-7 cells treated with hydrolysis products. However, the downregulation of these two cytokines may also have pro-tumorigenic effects.

(The figure was created with biorender.com).
