# Supplementary material for: Lipoprotein lipase hydrolysis products induce pro-inflammatory cytokine expression in triple-negative breast cancer cells
Source: BMC Res Notes. 2021 Aug 17;14:315. doi: 10.1186/s13104-021-05728-z (PMC8369739; doi:10.1186/s13104-021-05728-z)
Supplement: Supplementary file 4 — Additional file 4: Fig. S2. Expression of TNFA in MD-MBA-468 cells in response to the FFA component of total lipoprotein hydrolysis products generated by LPL. The FFA component that is generated from the hydrolysis of lipoprotein lipids by LPL was reconstituted as previously described—see Additional file 1: Detailed methodology. MDA-MB-468 cells were treated with either the FFA component or vehicle control for 18 h, as previously described—see Additional file 1: Detailed methodology. Following treatment, RNA was extracted from cells and examined for the expression of TNFA and normalized against the expression data for ACTB. Primer information and qPCR conditions were previously reported—see Additional file 1: Detailed methodology. Data are the average ± SD of two biological experiments. [file 13104_2021_5728_MOESM4_ESM.pdf]

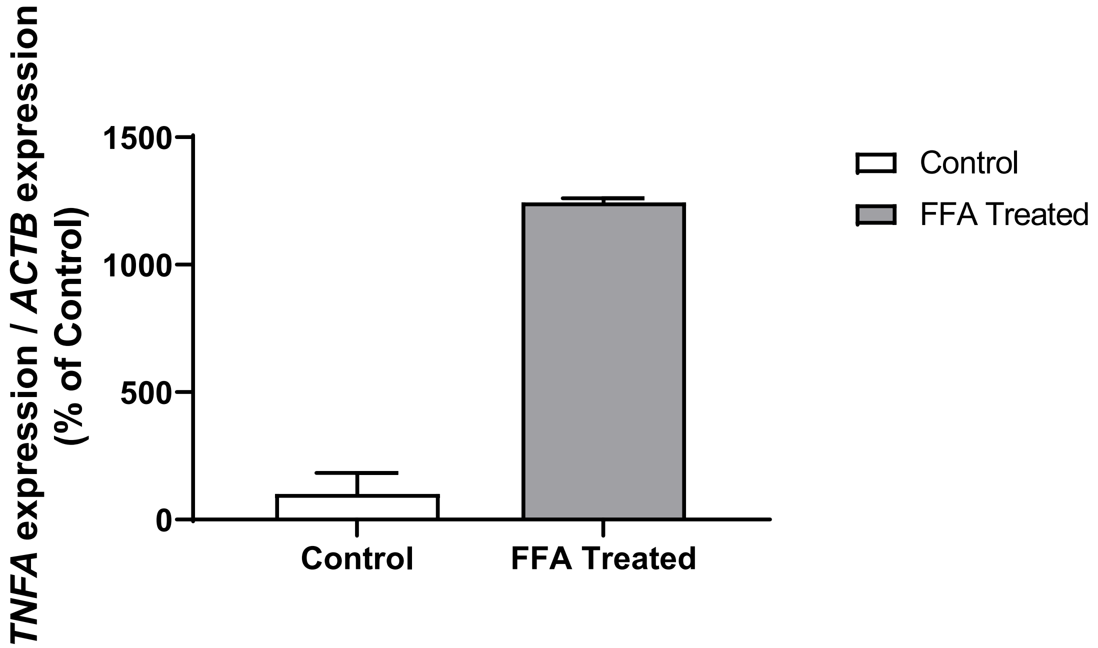

Figure S2. Expression of *TNFA* in MD-MBA-468 cells in response to the FFA component of total lipoprotein hydrolysis products generated by LPL. The FFA component that is generated from the hydrolysis of lipoprotein lipids by LPL was reconstituted as previously described – see Additional file 1: Detailed methodology. MDA-MB-468 cells were treated with either the FFA component or vehicle control for 18 h, as previously described – see Additional file 1: Detailed methodology. Following treatment, RNA was extracted from cells and examined for the expression of *TNFA* and normalized against the expression data for *ACTB*. Primer information and qPCR conditions were previously reported – see Additional file 1: Detailed methodology. Data are the average ± SD of two biological experiments.
